# Doping Ruthenium into Metal Matrix for Promoted pH‐Universal Hydrogen Evolution

**DOI:** 10.1002/advs.202200010

**Published:** 2022-03-25

**Authors:** Jiqing Jiao, Nan‐Nan Zhang, Chao Zhang, Ning Sun, Yuan Pan, Chen Chen, Jun Li, Meijie Tan, Ruixue Cui, Zhaolin Shi, Jiangwei Zhang, Hai Xiao, Tongbu Lu

**Affiliations:** ^1^ MOE International Joint Laboratory of Materials Microstructure Institute for New Energy Materials and Low Carbon Technologies School of Materials Science and Engineering Tianjin University of Technology Tianjin 300384 China; ^2^ Haihe Laboratory of Sustainable Chemical Transformations Tianjin 300192 China; ^3^ College of Materials Science and Engineering, State Key Laboratory of Bio‐Fibers and Eco‐Textiles Qingdao University Qingdao 266071 China; ^4^ Department of Chemistry Tsinghua University Beijing 100084 China; ^5^ State Key Laboratory of Heavy Oil Processing China University of Petroleum (East China) Qingdao 266580 China; ^6^ Dalian National Laboratory for Clean Energy & State Key Laboratory of Catalysis Dalian Institute of Chemical Physics Chinese Academy of Sciences Dalian 116023 P. R. China

**Keywords:** hydrogen evolution reaction, pH‐universal, surface engineering, zeolitic imidazolate frameworks

## Abstract

For heterogeneous catalysts, the active sites exposed on the surface have been investigated intensively, yet the effect of the subsurface‐underlying atoms is much less scrutinized. Here, a surface‐engineering strategy to dope Ru into the subsurface/surface of Co matrix is reported, which alters the electronic structure and lattice strain of the catalyst surface. Using hydrogen evolution (HER) as a model reaction, it is found that the subsurface doping Ru can optimize the hydrogen adsorption energy and improve the catalytic performance, with overpotentials of 28 and 45 mV at 10 mA cm^−2^ in alkaline and acidic media, respectively, and in particular, 28 mV in neutral electrolyte. The experimental results and theoretical calculations indicate that the subsurface/surface doping Ru improves the HER efficiency in terms of both thermodynamics and kinetics. The approach here stands as an effective strategy for catalyst design via subsurface engineering at the atomic level.

## Graphical abstract

Interface modulation via implanting ruthenium in matrix for improved hydrogen evolution performance in alkaline, acidic, and neutral media.

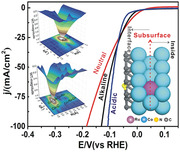

## 1 Introduction

Hydrogen (H_2_) is generally regarded as a promising renewable alternative to fossil fuels for its high energy density, environmental friendliness, and high abundance. Currently, the industrial hydrogen is produced primarily by coal gasification and steam reforming of methane, which usually contain poisoning agents (such as sulfides) and thus impede the utilization of hydrogen in fuel cells. Among the various approaches to H_2_ production, water electrolysis has been highly valued as a clean and sustainable method to produce H_2_ with high purity and large quantity.^[^ ^1^ ^]^ Great efforts have been devoted to developing high efficient catalysts for the hydrogen evolution reaction (HER) in water electrolysis, among which the noble‐metal‐based catalysts for HER have the advantages such as high catalytic activity, wide pH applicability, and good stability.^[^ ^2^, ^3^, ^4^, ^5^ ^]^ For instance, platinum (Pt) stands as the best‐performing catalyst for HER.^[^ ^6^ ^]^ However, the low abundances and high costs of noble metals are the critical factors that restrict their industrial‐scale applications.^[^ ^7^ ^]^ Non‐noble metal catalysts (in the form of nitrides, sulfides, phosphides, etc.), by contrast, have been considered as potential candidates by virtue of their abundant reserves, low costs, and potential catalytic performance; however, non‐noble metals have large overpotentials during HER and are prone to corrosion in acidic media.^[^ ^8^, ^9^, ^10^, ^11^, ^12^, ^13^ ^]^ As a new class of advanced catalysts, the single‐atom catalysts^[^ ^14^, ^15^ ^]^ have attracted enormous attention in HER for their maximized atom utilization and remarkable activity;^[^ ^4^, ^16^ ^]^ yet still, the disadvantages, such as low loading amounts, would hinder the overall HER performance. Therefore, the design of high‐efficiency and low‐cost catalysts for HER still presents as a major challenge for practical and economic H_2_ production in both acidic and alkaline media.

Cobalt (Co) is a common earth‐abundant transition metal,^[^ ^17^ ^]^ and ruthenium (Ru) is a relatively inexpensive noble metal with high corrosion resistance.^[^ ^18^, ^19^, ^20^, ^21^ ^]^ Yet the rather weak interaction between metallic Co and H is not conducive to the step of H adsorption in HER, whereas the overly strong binding between Ru and H atoms restrains the step of H_2_ desorption. It thus comes as a natural solution to engineer Co‐based alloys with Ru so as to tune the interfacial electronic states and the electrocatalytic properties,^[^ ^22^ ^]^ which may optimize the reversibility of H adsorption/desorption processes for improved HER performance.^[^ ^23^ ^]^ Lu et al. reported that Ru could induce lattice strain in the CoRu_0.5_ nanoalloys supported on carbon quantum dots, which displayed excellent catalytic activity for HER^[^ ^24^ ^]^; Chen et al. and Wang et al. separately reported CoRu nanoalloys encapsulated in nitrogen‐doped graphene^[^ ^3^ ^]^/carbon,^[^ ^25^ ^]^ which exhibited low overpotentials for HER. However, there are still unresolved key issues regarding the CoRu alloy catalysts for HER. First, the previously reported alloys all have a relatively high ratio of Ru.^[^ ^26^, ^27^ ^]^ Second, in these reported CoRu alloy catalysts, only the catalytic sites exposed on the surface were discussed, whereas the interaction exerted by the neighboring interior atoms was largely overlooked.^[^ ^26^, ^27^, ^28^ ^]^ Third, the precise elemental arrangement at the atomic level is far from clear, and the structure‐activity relationship is yet to be established.

Here, we develop a pyrolysis strategy to synthesize Co_x_Ru_y_ catalysts from CoRu‐based zeolitic imidazolate frameworks (ZIFs) with the assistance of melamine. In contrast to previous reports where the Ru atoms were inevitably exposed on the surface of CoRu alloy,^[^ ^29^ ^]^ our catalyst features low content of Ru prone to be doped in the subsurface of Co matrix. The catalyst has a core‐shell structure of Co_5_Ru_1_ particles encapsulated in N‐doped carbon nanotubes anchored on a polyhedral frame (NCNT/PF), which effectively enhances the activity in acidic, alkaline, and neutral media, and the catalyst even outperforms the commercial Ru/C for HER. The best‐performing catalyst Co_5_Ru_1_@NCNT/PF was obtained by tuning the atomic arrangement, which shows an optimized hydrogen adsorption energy for improved HER. The experimental results and density functional theory (DFT) calculations indicate that the Ru doped in the subsurface/surface of Co can boost the HER efficiency in terms of both thermodynamics and kinetics. Our work offers an effective strategy for catalyst design via surface engineering at the atomic level.

## 2 Results and Discussion

### 2.1 Preparation and Characterization of Ru Doped in Co Matrix Subsurface

**Figure 1:**
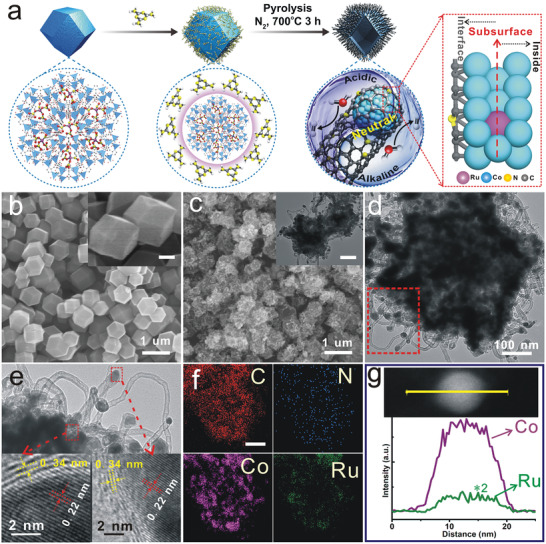
Structural characterizations of Ru doped in Co matrix subsurface (Co_5_Ru_1_@NCNT/PF). a) Schematic illustration of the fabrication procedure of encapsulated Co_5_Ru_1_@NCNT/PF. b) SEM images of CoRu‐based ZIF. c) SEM images of Co_5_Ru_1_@NCNT/PF; (inset, TEM image) d) TEM image of a single Co_5_Ru_1_@NCNT/PF particle. e) HRTEM image for different areas corresponding to the red box in (d). f) EDS mapping images for different elements (scale bar, 100 nm). g) Line‐scan profiles obtained with STEM‐EDS.

The preparation process of Ru doped in Co matrix subsurface is illustrated in **Figure** **1**a. First, ZIF‐67 containing Ru precursor was fabricated; the resulting dodecahedra were mixed with a measured amount of melamine (C_3_N_6_H_6_) under sonication in ethanol; then the mixture was subjected to pyrolysis at 700 °C for 3 h under N_2_ atmosphere to give the Co_5_Ru_1_@NCNT/PF catalyst. The molar ratio of Co/Ru was varied to obtain samples with different compositions.

The experimental details are summarized in the Supporting Information. The actual metal compositions of different Co_x_Ru_y_ alloys were determined by inductively coupled plasma mass spectroscopy (ICP‐MS), as listed in Table S1 (Supporting Information). X‐ray diffraction (XRD) patterns of different samples are shown in Figure S1 (Supporting Information). When the Ru content is very low, the samples of Co/C (No Ru) and Ru‐Co‐1 (Co_0.93_Ru_0.07_) showed XRD peaks typical of fcc phase (Co PDF# 15–0806). As the Ru content increases, the peak positions for Co_5_Ru_1_@NCNT/PF shift smoothly from hexagonal phases Co (PDF# 05–0727) to Ru (PDF# 06–0663).^[^ ^3^, ^30^ ^]^ Obviously, no Ru diffraction peaks can be found in XRD patterns of Co_5_Ru_1_@NCNT/PF, suggesting that the Ru atoms are incorporated into the Co lattice in the sample.^[^ ^30^, ^31^, ^32^ ^]^

The morphology and size of as‐synthesized samples are shown in Figure 1b–d. The scanning electron microscopy (SEM) image (Figure 1b) displays that the CoRu‐based ZIF before pyrolysis has a typical dodecahedral shape with a uniform size distribution ≈400 nm. After calcination, the surfaces of the polyhedral frames are covered with CNTs (Figure 1c). Figure 1d shows the transmission electron microscopy (TEM) image of a typical particle, in which encapsulated Co_5_Ru_1_@NCNTs are clearly demonstrated. And TEM images of different samples are shown in Figure S2 (Supporting Information). As shown in Figure 1e, the encapsulated structure of the alloy covered carbon was determined by the high‐resolution TEM (HRTEM) images from different selected areas. The lattice fringes with a spacing of 0.22 nm corresponds to the (100) facets of Co_5_Ru_1_; the lattice fringe with a spacing of 0.34 nm corresponds to the (002) facets of graphitic carbon planes.

The elemental distribution of the prepared catalyst was determined by energy‐dispersive X‐ray spectroscopy (EDS) mapping. Ru and Co were found to co‐exist on the alloy nanoparticles (Figure 1f), which is in agreement with the XRD data. The EDS line‐scan profiles of a random nanoparticle also confirm the co‐existence of Ru and Co throughout the alloy structure (Figure 1g). It is noteworthy that the profile of Ru signal was enveloped below that of Co, which gives us an important hint for the subsequent clarification of the atomic arrangement of Co and Ru.

X‐ray photoelectron spectroscopy (XPS) was conducted to probe the chemical states of related elements.^[^ ^33^, ^34^ ^]^ The Co 2p profile of Co_5_Ru_1_@NCNT/PF (Figure S3a, Supporting Information) showed the major 2p_3/2_ peaks at 778.4 and 781.5 eV that correspond to Co^0^ and Co^2+^, respectively.^[^ ^35^ ^]^ Compared with Ru‐free Co@NC, the Co 2p XPS peaks of Ru_x_Co_y_ alloy@NCNT/PF shifted to higher energy, which may result from the charge transfer from the less electronegative Co to the more electronegative Ru.^[^ ^36^ ^]^ The N 1s spectra (Figure S3b, Supporting Information) can be deconvoluted into three sub‐peaks that correspond to pyridinic N (398.8 eV), pyrrolic N (400.5 eV), and graphitic N (401.2 eV).^[^ ^37^, ^38^ ^]^ The C 1s signal (Figure S3c, Supporting Information) shows three main peaks at 284.7, 285.2, and 286.2 eV, which can be assigned to C═C, C═N, and N‐doped carbon (C—N), respectively.^[^ ^31^ ^]^

### 2.2 XAFS Analysis of Ru Doped into Co Matrix Subsurface

**Figure 2:**
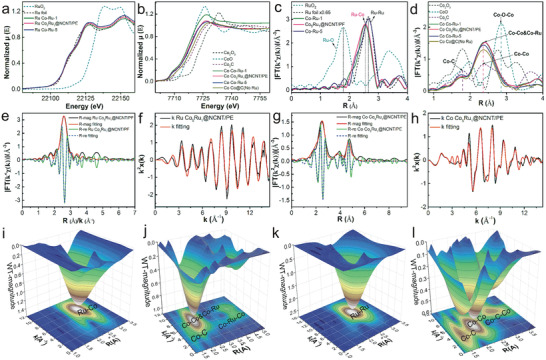
XAFS of Ru K‐edge and the Co K‐edge for Ru doped in Co matrix subsurface and other samples. a,b). Normalized XANES *χ*(E) spectra of Ru and Co, with Ru foil, RuO_2_, CoO, Co_2_O_3_, and Co_2_C as references, respectively. c,d) Radial distance *χ*(R) space spectra of Ru and Co. (e) and (g) *χ*(R) space spectra fitting curve of Ru and Co for Co_5_Ru_1_@NCNT/PF. f,h) *χ*(k) space spectra fitting curve of Ru and Co for Co_5_Ru_1_@NCNT/PF. i–l) wavelet transform of *χ*(k) of Ru and Co Co_5_Ru_1_@NCNT/PF, Ru foil, and Co_2_C.

To investigate the atoms arrangements, valence states and coordination environments of Ru and Co atoms in different samples, we conducted synchrotron‐radiation‐based X‐ray absorption fine structure (XAFS) at the Ru K‐edge and the Co K‐edge for different Co_x_Ru_y_ samples, and the corresponding references including Ru foil, RuO_2_, CoO, Co_2_O_3_, and Co_2_C. Three Co_x_Ru_y_ catalysts prepared with different Co/Ru ratios were selected and investigated, namely, the best catalyst Co_5_Ru_1_@NCNT/PF, Co‐Ru‐1(Co:Ru = 12:1), and Co‐Ru‐5 (Co:Ru = 3:2). The oxidation state of the metal in a statistical nature can be analyzed from the X‐ray absorption near edge structure (XANES) region on the basis of the edge position and white line intensity. As shown in **Figure** **2**a, the profiles for the three Co_x_Ru_y_ catalysts are similar to that of Ru foil, yet Co‐Ru‐5 shows a slight increase in white line intensity; for all three Co_x_Ru_y_ catalysts, no shifts in edge position were observed in reference to RuO_2_. It revealed a slightly oxidized state of Ru in Co‐Ru‐5 relative to the Ru foil. As for the Co K‐edge signals, the edge absorption energies of the three Co_x_Ru_y_ catalysts are slightly shifted toward that of CoO, and the white line intensities are significantly increased compared with that Co@CN (No Ru) (Figure 2b). These data indicate an oxidized state of Co in the prepared alloys. The above results are consistent with the XPS data (Figure S3, Supporting Information). For XANES, the Co displayed a high oxidation state because of surface oxidation and electron transfer from Co to Ru (as Ru has a higher electronegativity than Co).

The extended X‐ray absorption fine structure (EXAFS) was employed to investigate the local atomic structure and to deduce the structural parameters such as coordination numbers, bond lengths, and the extent of alloying. As shown in Figure 2c, for Co‐Ru‐5, the weak signal of Ru–O at 1.82 Å in the first coordination shell indicates that the Ru atoms were slightly oxidized, which revealed that some Ru atoms were exposed on the surface. By contrast, the very weak Ru–O signal was distinguished in Co_5_Ru_1_@NCNT/PF, which suggests that at lower Ru contents, the Ru were prone to be implanted into the Co matrix rather than to be exposed on the surface. The deduction can be confirmed by EXAFS of Co‐Ru‐1, the Ru–O signals were not found in the lowest content of Ru. Notably, the radial distance space spectra *χ*(R) for the Ru of Co_5_Ru_1_@NCNT/PF and Co‐Ru‐1 showed a major peak at ≈2.57 Å, which is attributed to the Ru–Co scattering path in the second coordination shell, indicating that the Ru atoms were dispersed in the alloy catalysts. And the Ru–Co path is slightly shorter than the Ru–Ru path (≈2.65 Å) owing to the lattice strain,^[^ ^24^ ^]^ suggesting that the lots of Ru atoms are incorporated into the Co subsurface lattice. The same conclusion can also be drawn from the *χ*(R) data for Co K‐edge (Figure 2d, and the HRTEM images in Figure S4, Supporting Information). By contrast, the primary peak of Co‐Ru‐5 shifted to a longer distance, locating at almost the same position for Ru–Ru bonds for Ru foil. These data indicate that the metallic Ru phase was formed in Co‐Ru‐5 owing to the elevated Ru content. Such an inference was further verified by the *χ*(R) for Co K‐edge of Co‐based samples. A common peak ≈1.77 Å was found for all these samples, which is attributed to Co–C scattering path (as in Co_2_C) in the first coordination shell, indicating that the surface Co atoms are coordinated with N‐doped carbon. Thus, it can be inferred that the Ru in Co_5_Ru_1_@NCNT/PF were most likely doped in the second coordination shell, rather than the first coordination shell.

Quantitative *χ*(R) and *χ*(k) space spectra fitting were also performed to investigate the local atomic structure and to further obtain the coordination numbers of Ru or Co in Co_5_Ru_1_@NCNT/PF (Figure 2e–h). Both the first and the second coordination shell were taken into consideration in Co and Ru *χ*(R) space spectra fitting for the Co‐based samples (Figures S5–S9, Supporting Information). The combination of *χ*(R) and *χ*(k) space spectra fitting was an intuitive way to distinguish such merged different scattering path signal contributions from each other. The Co_5_Ru_1_@NCNT/PF displayed Ru–Co bonding with a coordination number (CN) close to 8.0 (CN = N*amp = 10.0*(0.80 ± 0.15)) at 2.57 Å, whereas Co‐Ru‐5 displayed a decreased Ru–Co bonding at 2.65 Å, with a CN close to 5.0 (CN = N*amp = 6.0*(0.82 ± 0.17)). Similarly, in Co K‐edge *χ*(R) space spectra, Co_5_Ru_1_@NCNT/PF revealed Ru–Co bonding with a CN near 4.0 (CN = N*amp = 4.0*(0.78 ± 0.20)) at 2.60 Å, whereas Co‐Ru‐5 displayed Ru–Co bonding with a CN near 2.0 (CN = N*amp = 2.0*(0.78 ± 0.20)) at 2.65 Å. The quantitative *χ*(R) space spectra and CN data for the other samples can be found in Tables S2 and S3 (Supporting Information). A good fitting result was obtained from the *χ*(R) and *χ*(k) space spectra of Co_5_Ru_1_@NCNT/PF with a reasonable R factor. Furthermore, the wavelet transform (WT) of *χ*(k) is an intuitive way to demonstrate the degrees of alloying in Co_5_Ru_1_@NCNT/PF in comparison to the corresponding Ru foil and Co_2_C reference as shown in Figure 2i–l. For the Ru K‐edge WT of *χ*(k) spectra of Co_5_Ru_1_@NCNT/PF, only one merged scattering path signal of Ru–Co bond located at (*χ*(k), *χ*(R)) of (9.4, 2.60) was found. Compared with the characteristic scattering path signal of Ru–Ru bond for Ru foil (located at (9.8, 2.65)), the increased distance in *χ*(R) of Co_5_Ru_1_@NCNT/PF was due to the lattice strain induced by doping Ru, whereas the heterometallic bonding with the lighter Co element has resulted in the decrease in *χ*(k). The signal of Ru–O scattering path was not observed, indicating that a major portion of Ru were prone to be doped into the Co matrix rather than to be exposed on the surface. For the Co K‐edge WT of *χ*(k) spectra of Co_5_Ru_1_@NCNT/PF, all the distinguished scattering path signals of Co–C, Co–Co, and Co–Ru, Co–Ru–Co bonds located respectively at (4.0, 1.75), (7.4, 2.39), and (8.0, 4.72) from the first to third coordination shell can be observed. This indicates that the Co atoms were distributed from the surface to the inner bulk phase in Co_5_Ru_1_@NCNT/PF. To sum up, the XAFS analysis clearly revealed the atomic arrangements for Co_5_Ru_1_@NCNT/PF, in which the lots of Ru atoms were prone to be dispersed at the subsurface of Co matrix, and the electronic structure of catalyst surface was thus engineered by the Ru doping.

### 2.3 Assessment of HER Performance

**Figure 3:**
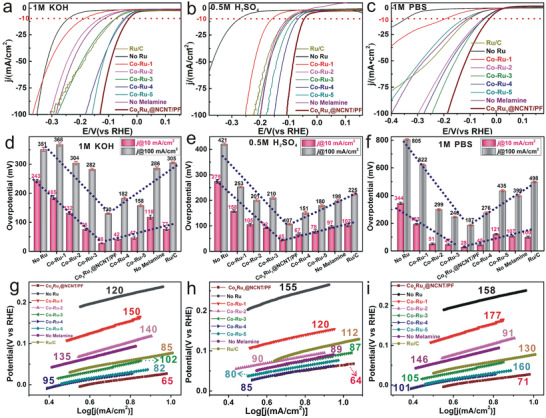
Catalytic performances of Ru doped in Co matrix subsurface and other samples in HER. a–c) The LSV polarization curves, d–f) overpotentials at the current density of 10 and 100 mA cm^−2^, g–i) Tafel plots of catalyst in N_2_‐saturated 1 m KOH, 0.5 m H_2_SO_4_, and 1 m PBS solutions, respectively.

In order to unravel the key factors that contribute to the electrocatalytic HER performance of Co‐based catalysts, we compared a series of samples. Aside from Co_5_Ru_1_@NCNT/PF, five samples with different Co/Ru nominal ratios were also prepared (denoted as Co‐Ru‐*x*, where *x* = 1–5; see Supporting Information for details). We also prepared a sample without Ru precursor, and a sample without melamine introduced. In addition, commercial Ru/C was also investigated as a comparison sample. All the catalysts were assessed in N_2_‐saturated alkaline (1 m KOH), acidic (0.5 m H_2_SO_4_) and neutral (1 m PBS) solutions using a standard three‐electrode setup. The linear sweep voltammetry (LSV) polarization curves in **Figure** **3**a–c were recorded with a scan rate of 5 mV s^−1^. Notably, the Co_5_Ru_1_@NCNT/PF catalyst displays the highest HER activity among all the catalysts reported thus far, giving the smallest overpotentials of 28, 45, and 28 mV (at a current density of 10 mA cm^−2^) in alkaline, acidic, and neutral media, respectively (Figure 3d**–**f), and the Tafel slopes for HER over Co_5_Ru_1_@NCNT/PF catalyst are also the smallest, with the values of 65, 64,and 71 mV dec^−1^ in alkaline, acidic, and neutral media, respectively (Figure 3g**–**i). It is worth mentioning that the HER performance of our Co_5_Ru_1_@NCNT/PF catalyst is even better than that of the commercial Ru/C (overpotentials of 77, 102, and 100 mV at 10 mA cm^−2^, and Tafel slopes of 85, 112, and 130 mV dec^−1^ in alkaline, acidic, and neutral media, respectively). In particular, at a higher current density of 100 mA cm^−2^, the Co_5_Ru_1_@NCNT/PF gives the lowest overpotentials (128, 108, and 187 mV in alkaline, acidic, and neutral media, respectively) among all the catalysts. To the best of our knowledge, in neutral electrolyte, our catalyst gives the smallest overpotential reported thus far. Compared with previous reports (Table S4, Supporting Information), the best‐performing sample Co_5_Ru_1_@NCNT/PF displayed high activities. Notably, in neutral media the overpotential at 10 mA cm^−2^ is the smallest among recently reported data (Table S5, Supporting Information).

#### 2.3.1 The Effect of Melamine as an Additive

When no melamine was added during the synthesis, aggregated frame particles were obtained, with barely any encapsulated structure found on the surface (Figure S10, Supporting Information), and the resulting Co_5_Ru_1_ catalyst gives higher overpotentials (113, 95, and 107 mV) and higher Tafel slopes (135, 89, and 146 mV dec^−1^) than Co_5_Ru_1_@NCNT/PF in alkaline, acidic, and neutral media. The electrochemically active surface area (ECSA) was determined using the double‐layer capacitance (*C_dl_*) method, and the results show that the encapsulated structure has a larger ECSA (Figure S11, Supporting Information).

#### 2.3.2 The Effect of Ru Content

The overpotentials for different catalysts are summarized in Figure 3c,d, in which the catalysts are ranked along the horizontal axis according to the Ru content. The typical inverted‐volcano plots were found. The overpotentials of Ru‐free catalyst are quite large (243, 275, and 344 mV at 10 mA cm^−2^ in alkaline, acidic, and neutral media, respectively). The best‐performing Co_5_Ru_1_@NCNT/PF catalyst was prepared with a Ru/Co nominal molar ratio of 1:2. When the Ru/Co nominal ratio was increased to 3:4 and 1:1, the overpotentials became higher (37 and 42 mV in alkaline media, 68 and 79 mV in acidic media). These data confirmed that the Ru content is a key factor for the HER performance. In the next section, we try to unveil the precise atomic arrangement of Ru and Co in the alloy matrix.

### 2.4 Theoretical Modeling

To further elucidate the roles of contributing factors, we performed DFT based modeling of HER on six catalyst models: pristine Co(0001) and pristine Ru(0001), RuCo(0001) in which one Ru atom is either placed on the surface acting as a single‐atom alloy or doped in the subsurface of Co matrix, and graphitic N‐doped graphene (C/gN) supported RuCo(0001) with Ru doping in the aforementioned same ways. Table S6 (Supporting Information) displays the free energies for HER featuring the only key intermediate, i.e., the adsorbed *H. Note that the *H that we considered here is at the top site, as it has been shown in a previous study to be the active species (whereas the *H at the hollow site serves as a spectator).^[^ ^39^ ^]^ Co(0001) surface does not favor *H binding, with the free energy uphill by 0.26 eV, indicating a potential‐limiting Volmer step, whereas the Ru(0001) surface presents a virtually optimal *H adsorption free energy downhill by only 0.06 eV. On the RuCo(0001) surface with Ru implanted in the subsurface either with or without C/gN support, *H prefers to bind those surface Co sites nearest to the subsurface Ru, with an adsorption free energy of 0.14 or 0.20 eV. Although such a case is ostensibly only suboptimal with respect to the case where Ru is present at the surface, overall it can be concluded that introducing Ru (either at the surface or the subsurface) can facilitate *H binding, thus demonstrating the catalytic power bestowed by Ru upon the Co surface. DFT results show that the Ru doping in the subsurface, even at a low concentration, dramatically improves the catalytic activity for HER. In addition, it is noteworthy that the model with Ru doped in the subsurface is thermodynamically more stable (by 0.04 eV) than that with Ru alloyed in the surface.

**Figure 4:**
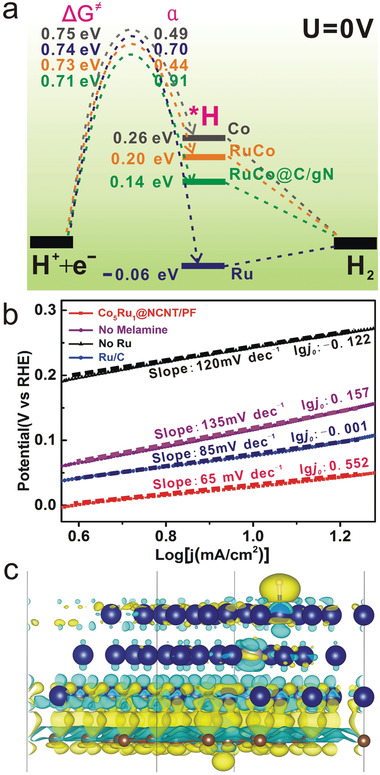
Theoretical model for Ru doped in Co matrix subsurface. a) The free energy diagrams for HER on Co (0001), Ru (0001), CoRu (0001), and C/gN‐supported CoRu (0001) surfaces. b) Experimental Tafel plots for HER on different catalysts. c) The charge density difference in C/gN‐supported RuCo(0001) surface with one Ru atom substituting one Co atom in the subsurface upon H adsorption.(C: brown; N: light blue; Co: navy blue; Ru: gray; H: white. The contour value of the charge density is 0.0025 a.u. The charge accumulation regions are in yellow color while the charge depletion regions are in cyan color).

Yet still, the above analysis from the thermodynamic perspective does not explain the fact that RuCo@NCNT/PF is the best‐performing catalyst (even significantly better than the commercial Ru/C catalyst), and thus we resorted to the analysis on the kinetics. Using a recently proposed approach based on the Tafel approximation to the generalized Butler–Volmer equation,^[^ ^40^ ^]^ we derived the free energy barrier (ΔG^≠^) and electron transfer coefficient (*α*) for the Volmer step on each catalyst model from the experimental Tafel slopes (**Figure** **4**a; Table S7, Supporting Information). Note that the calculated free energy barriers correspond to the kinetics of the rate‐determining step of HER in alkaline and neutral conditions, i.e., the Volmer step coupled with the dissociation of H_2_O (* + H_2_O + e^−^ = *H + OH^−^). From the kinetic perspective, the RuCo(0001)@C/gN model indeed renders the lowest barrier (0.71 eV) among all the models, and the values of *α* are even more revealing: the RuCo(0001) without C/gN has an *α* value (0.44) similar to that for Co (0.49), while RuCo@C/gN has a much higher *α* value (0.91), even higher than that for Ru (0.70). Previous studies have reported that a suitable amount of N doping could further enhance the HER activity.^[^ ^13^, ^41^ ^]^ Figure S3b (Supporting Information) shows that the graphitic N content increases from 23.5% in the Co_5_Ru_1_ catalyst (prepared without melamine) to 28.6% in the optimized Co_5_Ru_1_@NCNT/PF catalyst. From Figure S12 (Supporting Information), the Nyquist plot^[^ ^11^ ^]^ displays a better charge transferability for the encapsulated structure with NCNT/PF resulting from the melamine‐assisted synthesis. We found that the presence of graphitic N is correlated with the improvement of HER performance. The graphitic N can donate electrons to the alloy catalyst, making it electron‐rich and thus promoting the electron transfer at the interface during HER.^[^ ^42^ ^]^ Therefore, the C/gN support plays an important role in improving the electron transfer kinetics for the Volmer step on the catalyst (Figure 4b). Figure 4c shows that upon *H adsorption, the C/gN support loses electrons through the contact region to the alloy, and the electrons may migrate to the alloy surface to reduce protons. To sum up, doping Ru into the subsurface improves the *H adsorption free energy thermodynamically, while incorporating the C/gN support promotes the electron transfer kinetically, and it is the combination of these two factors that result in the optimal performance of RuCo@NCNT/PF for HER.

## 3 Conclusion

In summary, we reported a surface‐engineering strategy to dope Ru into the subsurface/surface of Co matrix, which tuned the electronic structure and lattice strain of the catalyst surface, and thus optimized the hydrogen adsorption energy for improved HER. Compared with previous reports, the best‐performing sample Co_5_Ru_1_@NCNT/PF displayed high activities (with overpotentials of 28, 45, and 28 mV at 10 mA cm^−2^ in 1.0 m KOH, 0.5 m H_2_SO_4_, and 1.0 m PBS, respectively). Notably, in neutral media the overpotential at 10 mA cm^−2^ is the smallest among recently reported data. The XAFS analysis clearly revealed the atomic arrangements in Co_5_Ru_1_@NCNT/PF, in which the lots of Ru were prone to be dispersed at the subsurface of Co matrix, and the electronic structure of catalyst surface was thus engineered by the Ru doping. The experimental results and DFT calculations indicated that the subsurface/surface‐doped Ru could improve the HER efficiency in terms of both thermodynamics and kinetics. Our approach here stands as an effective strategy for catalyst design via surface engineering at the atomic level.

## 4 Experimental Section

Detailed experimental procedures can be found in the Supporting Information.

## Conflict of Interest

The authors declare no conflict of interest.

## Supporting information

Supporting Information

## Data Availability

The data that support the findings of this study are available in the supplementary material of this article.
