# Supplementary material for: Doping Ruthenium into Metal Matrix for Promoted pH‐Universal Hydrogen Evolution
Source: Adv Sci (Weinh). 2022 Mar 25;9(15):2200010. doi: 10.1002/advs.202200010 (PMC9130909; doi:10.1002/advs.202200010)
Supplement: Supplementary file 1 — Supporting Information [file ADVS-9-2200010-s001.pdf]

ADVANCED SCIENCE
Open Access

Supporting Information

for *Adv. Sci.*, DOI 10.1002/advs.202200010

Doping Ruthenium into Metal Matrix for Promoted pH-Universal Hydrogen Evolution

Jiqing Jiao, Nan-Nan Zhang, Chao Zhang, Ning Sun, Yuan Pan, Chen Chen, Jun Li, Meijie Tan, Ruixue Cui, Zhaolin Shi, Jiangwei Zhang*, Hai Xiao* and Tongbu Lu**

((Supporting Information can be included here using this template))

Supporting Information

Doping ruthenium into metal matrix for promoted pH-universal hydrogen evolution

Jiqing Jiao, Nan-Nan Zhang, Chao Zhang, Ning Sun, Yuan Pan, Chen Chen, Jun Li, Meijie Tan, Ruixue Cui, Zhaolin Shi, Jiangwei Zhang*, Hai Xiao* and Tongbu Lu**

This Supporting Information contains:

1. Experimental Procedures

Materials

HER electrochemical tests

Characterization

XAFS analysis

2. Computational Details

3. Supporting Figures

4. Supporting Tables

5. Supporting References

1. Experimental Procedures

Materials

$Co(NO_3)_2{\cdot}6H_2O$, ruthenium(III) nitrosyl nitrate, 2-methylimidazole, melamine($C_3H_6N_6$), ethanol, KOH, H_2SO_4 were purchased from Sinopharm Chemical Reagent Co. Ltd. The commercial Ru/C catalyst was purchased from Sigma-Aldrich (product number: 738549-1G). All the chemicals used were analytical grade and were not further purified.

Preparation of Co_5Ru_1@N-doped carbon nanotube polyhedral frames (NCNT/PF) by melamine-assisted pyrolysis

$Co(NO_3)_2{\cdot}6H_2O$ (1.16 g, 4.0 mmol) was dissolved in 100.0 mL methanol. 2-Methylimidazole (1.31 g, 16.0 mmol) was dissolved in 100.0 mL methanol, and then the solution of 2-methylimidazole was poured into the solution of $Co(NO_3)_2{\cdot}6H_2O$. After mixing and stirring at room temperature for 24 h, the prepared ZIF-67 was separated by centrifugation and washed with methanol three times. And then the ZIF-67 dodecahedra were dispersed in 10 mL ethanol, and ruthenium(III) nitrosyl nitrate was added to the above solution under ultrasonication for 30 min. The Ru-ZIF-67 was obtained after stirring for 3 h. The dry Ru-ZIF-67 powder (0.40 g) and melamine (1.20 g) were mixed in ethanol under sonication for 30 min, and the solid was collected by centrifugation, washed three times with ethanol and dried in vacuum at 60 °C for 6 h. At last, the pre-sample was calcined at 700 °C under N_2 flow for 3 h with a ramp rate of 2 $°C{\cdot}min^{-1}$.

Other Co-based catalysts were prepared by various nominal weight ratios of ZIF-67/ruthenium(III) nitrosyl nitrate.

XAFS measurements

The Ru K-edge X-ray absorption fine structure spectra were collected at BL14W1 beamline of Shanghai Synchrotron Radiation Facility (SSRF) while Co K-edge spectra were collected at 1W1B beamline of Beijing Synchrotron Radiation Facility (BSRF). The data were collected in fluorescence mode using a Lytle detector while the corresponding reference samples were collected in transmission mode. The samples were ground and uniformly daubed on the special adhesive tape.

The acquired EXAFS data were processed according to the standard procedures using the ATHENA module of Demeter software packages.

XAFS analysis

The acquired EXAFS data were processed according to thestandard procedures using the ATHENA module of Demeter software packages.

The EXAFS spectra were obtained by subtracting the post-edge background from theoverall absorption and then normalizing with respect to the edge-jump step. Subsequently, theχ(k) data of were Fourier transformed to real (R) space using a hanning windows (dk=1.0 $Å^{-1}$) toseparate the EXAFS contributions from different coordination shells. To obtain the quantitativestructural parameters around the central atoms, least-squares curve parameter fitting was performedusing the ARTEMIS module of Demeter software packages.

The following EXAFS equation was used:

$$\chi(k) = \sum_j \frac{N_j S_0^2 F_j(k)}{kR_j^2} \cdot \exp[-2k^2\sigma_j^2] \cdot \exp[\frac{-2R_j}{\lambda(k)}] \cdot \sin[2kR_j + \phi_j(k)]$$

the theoreticalscattering amplitudes, phase shifts and the photoelectron mean free path for all paths calculated. S_0^2 is the amplitude reduction factor, $F_j(k)$ is the effective curved-wave backscattering amplitude, N_j is the number of neighbors in the jth atomic shell, R_j is the distance between the X-ray absorbing central atom and the atoms in the jth atomic shell (backscatterer), λ is the mean free path in Å, $\phi_j(k)$ is the phase shift (including the phase shift for each shell and the total central atom phase shift), σ_j is the Debye-Waller parameter of the jth atomic shell (variation of distances around the average R_j). The functions $F_j(k)$, λ and $\phi_j(k)$ were calculated with the ab initio code FEFF9. The additional details for EXAFS simulations are given below.

All fits were performed in the *R* space with *k*-weight of 2 while phase correction was also applied in the first coordination shell to make R value close to the physical interatomic distance between the absorber and shell scatterer. The coordination numbers of model samples were fixed as the nominal values. While S_0^2, the internal atomic distances R, Debye-Waller factor σ^2, and the edge-energy shift Δwere allowed to run freely.

HER electrochemical tests

The prepared catalysts (5.0mg) were first dispersed in 1 mL ethanol/ Nafion (20 μL) solution and sonicated for 30 mins to obtained the ink. A certain volume of the catalyst ink was dropped onto the surface of GCE with Co_5Ru_1@CNT loading of 0.450 mg cm^{-2} and the loading of Ru/C 0.102 mg cm^{-2}, and drying naturally.All the measurements were carried out on a CHI 760E electrochemical workstation (CH Instruments, Inc., Shanghai) with a standard three-electrode setup. A glassy carbon electrode (GCE, 4 mm in diameter) was used as the support for the working electrode. Ag/AgCl electrode in saturated KCl l (3.5 M) and graphite rod were served as the reference electrode and counter electrode, respectively. The HER

performance was evaluated in N_2-saturated 1 M KOH, 0.5 M H_2SO_4 and 1M PBS solutions, respectively. All the potentials were convert to the reversible hydrogen electrode (RHE) by equation.

$$\mathrm{V_{RHE}} = \mathrm{E}_{(VS\ Ag/AgCl)} + 0.197\ + 0.0592 * pH$$

Linear sweep voltammetry (LSV) plots were recorded by applying proper potential ranges at a scan rate of 10 mV/s, and the Tafel plots were recorded at the scan rate of 1 mV/s at proper potential ranges.TheTafel slopes were obtained from the Tafel equation: (η=a +$blog\ j$, where b is the Tafel slope) in a linear region of the Tafel plots.

Characterization

X-ray diffraction (XRD) was carried out with a Rigaku D/max 2500Pc X-ray powder diffractometer with monochromatized Cu Kα radiation (λ =1.5418 Å). HRTEM, HAADF-STEM and the line-scan analysis were carried out on a JEOL JEM-2100F field emission electron microscope working at 200 kV. X-ray photoelectron spectroscopy (XPS) was operated with Thermo Fisher (ESCALAB 250Xi). SEM images were obtained on a Hitachi S-4800 electron microscope. TEM images were obtained by a Hitachi-7700 working at 100 kV.The concentrations of elements were measured with a Thermo Scientific PlasmaQuad 3 inductively-coupled plasma mass spectrometry (ICP-MS) after dissolving the samples with a mixture of HCl and HNO_3 (3:1, volume ratio).

2. Computational Details

The optimization of structures was performed with the VASP package,[1-3] using the Perdew-Burke-Ernzerhof (PBE) approximation of density functional theory (DFT) and the projector augmented wave (PAW) method to account for core-valence interactions.[4,5] The kinetic energy cutoff for plane wave expansions was set to 400 eV, and the reciprocal space was sampled by a 3×3×1 grid. The convergence criterion is 1×10^{-5} energy difference for solving for the electronic wave functions. All atomic coordinates are converged to within 3×10^{-2} eV/Å for maximal components of forces. The zero-point energies (ZPE) and enthalpy and entropy contributions to free energies at room temperature (298.15 K) were included ignoring the two imaginary frequencies vibrating in surface plane resulting from the top site *H we postulated, which will be discussed later.

The slab models for Co(0001) and Ru(0001) were constructed using the experimental lattice parameters with 4×4 surface supercells and 3 layers.[6-8] Co(0001) slab was modified further with one Ru atom substituting one Co atom in the surface or subsurface to build the corresponding RuCo(0001) models. The C/gN support was introduced at the bottom as a 4×4 graphene monolayer doped with one graphitic N atom. All atoms were subject to relaxation. Vacuum layers of more than 15 Å were introduced to eliminate any artificial interactions between periodic images along the *c* axis.

As the *H we considered is at the top site, we found that this type of structure has two imaginary frequencies, which are 329 cm^{-1} and 364 cm^{-1} along *a* and *b* axis in the case of (Subsurface)RuCo@C/gN when fixing all atoms but the only *H. Based on this surface, we built the model taking the coverage into consideration, in which three neighboring *fcc* hollow sites surrounding the top site *H are all filled with *H. As we expected, no imaginary frequency appears. Thus we speculate that in real circumstances with *H filling the hollow sites in an energetically more favorable way and acting as the spectator, top site is a

metastable state and can truly exist. So it is reasonable to consider just the top site as the active site as a simplification.

3. Supporting Figures

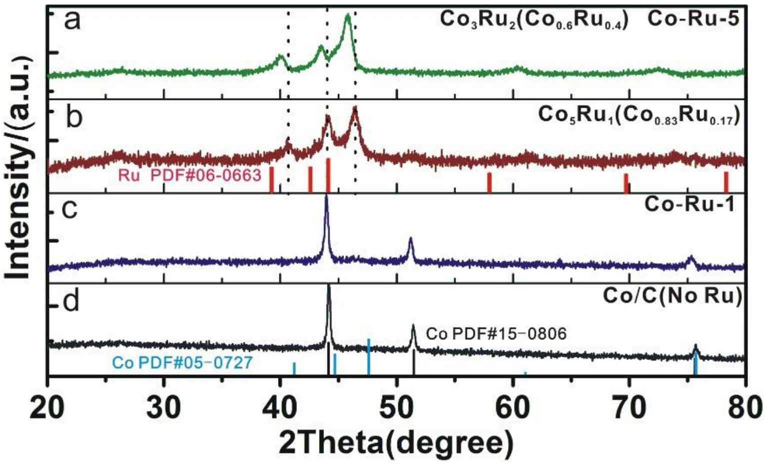

Figure S1. XRD patterns of different samples. (a) Co-Ru-5, (b) Co_5Ru_1@NCNT/PF, (c) Co-Ru-5 and (d) No Ru

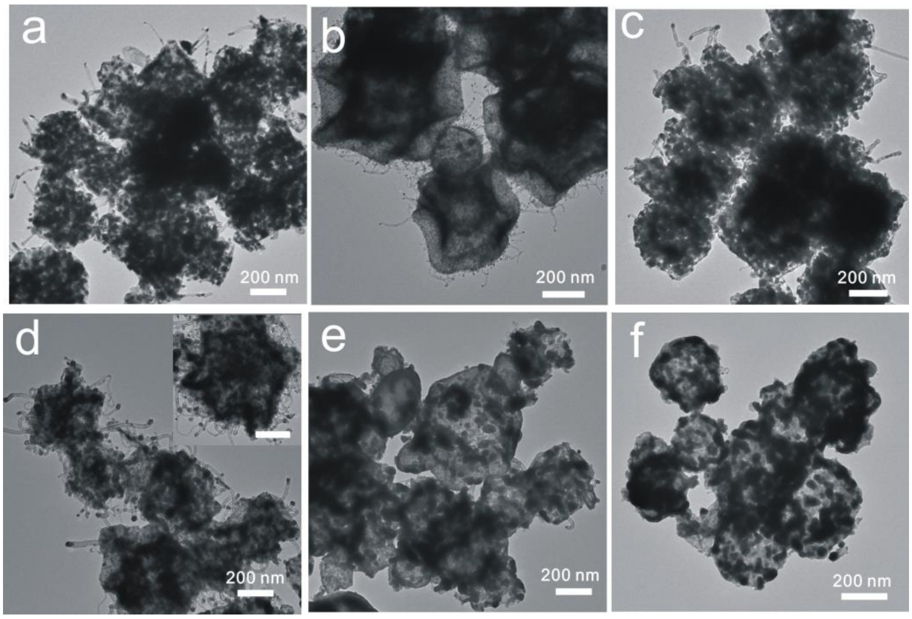

Figure S2. TEM images of samples with various nominal ratios of ZIF67: Ru(III). (a) Co-Ru-1, (b) Co-Ru-2, (c) Co-Ru-3, (d) Co_5Ru_1@NCNT/PF, (e) Co-Ru-4 and (f)Co-Ru-5.

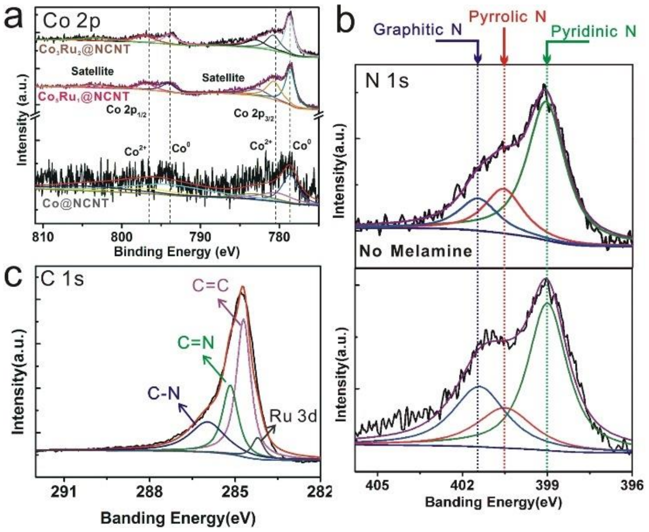

Figure S3. (a) High resolution XPS spectrum of Co 2p for Co_5Ru_1@NCNT/PF, Co_3Ru_2@NCNT/PF and Co@NCNT/PF. (b) High resolution XPS spectra of N 1s for Co_5Ru_1 and Co_5Ru_1@NCNT/PF. (c) High resolution XPS spectrum of C 1s for Co_5Ru_1@NCNT/PF.

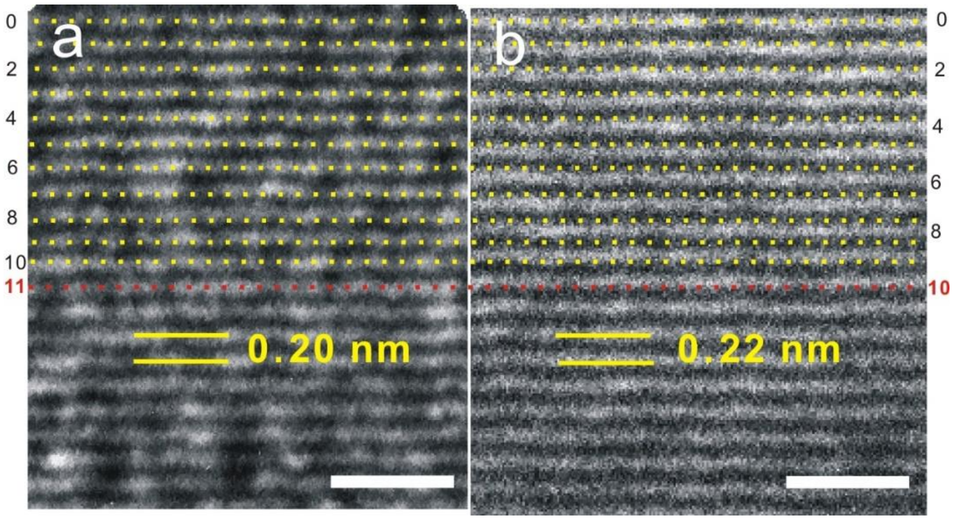

Figure S4. HRTEM images of (a) Co@CN (No Ru) and Co_5Ru_1@NCNT/PF.

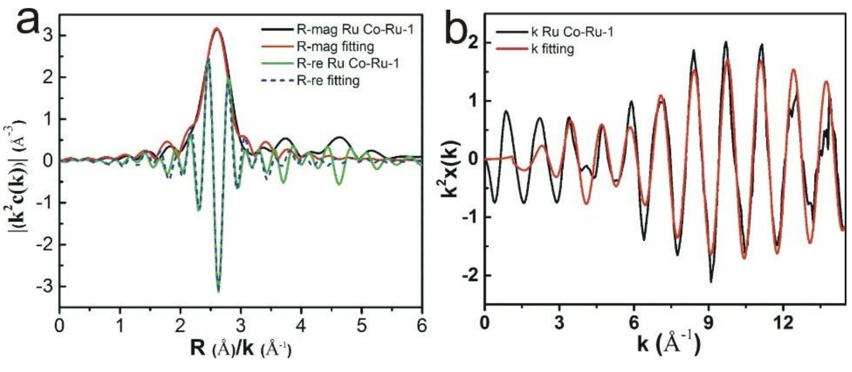

Figure S5. (a) χ(R) and (b) χ(k) space spectra fitting curve of Ru for Co-Ru-1.

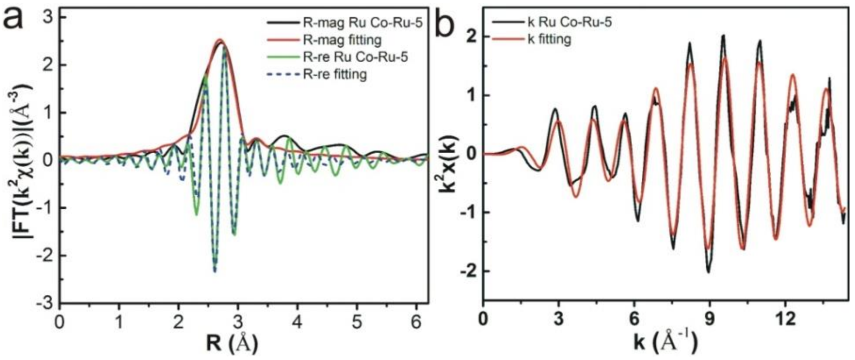

Figure S6. (a) χ(R) and (b**)** χ(k) space spectra fitting curve of Ru for Co-Ru-5.

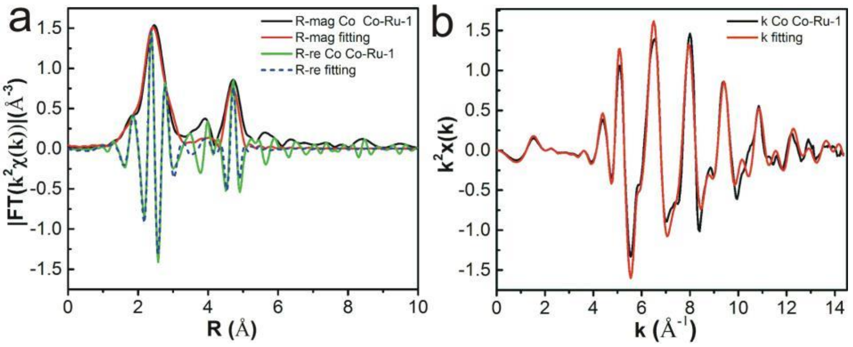

Figure S7. (a) χ(R) and (b) χ(k)space spectra fitting curve of Co for Co-Ru-1

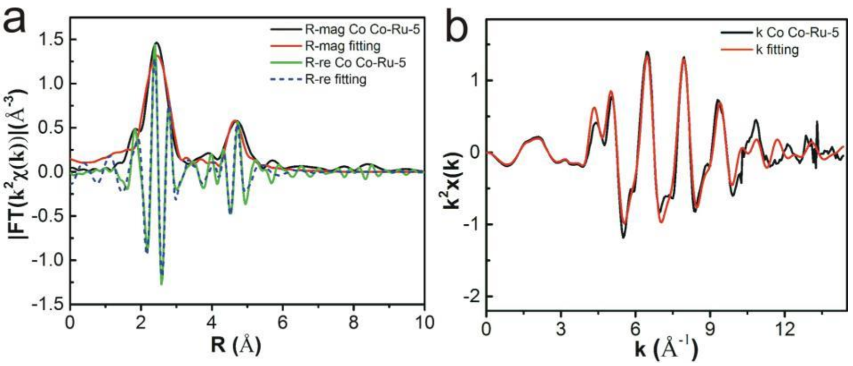

Figure S8. (a) χ(R) and (b) χ(k) space spectra fitting curve of Co for Co-Ru-5

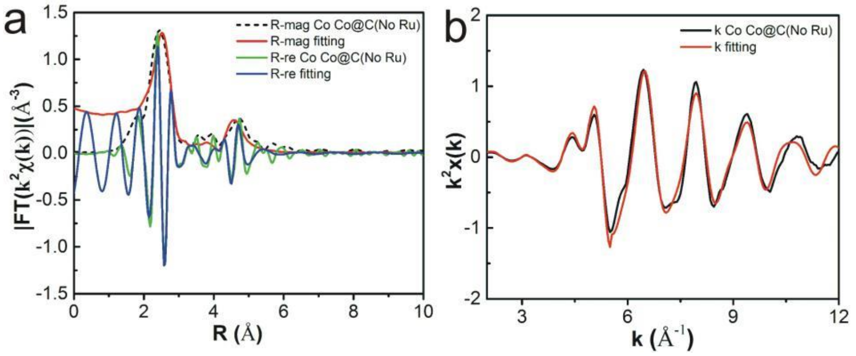

Figure S9. (a) χ(R) and (b) χ(k) space spectra fitting curve of Co for catalyst (No Ru)

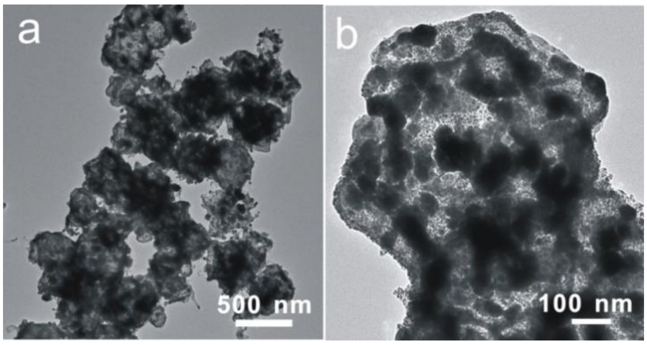

Figure S10. TEM images of catalyst without melamine.

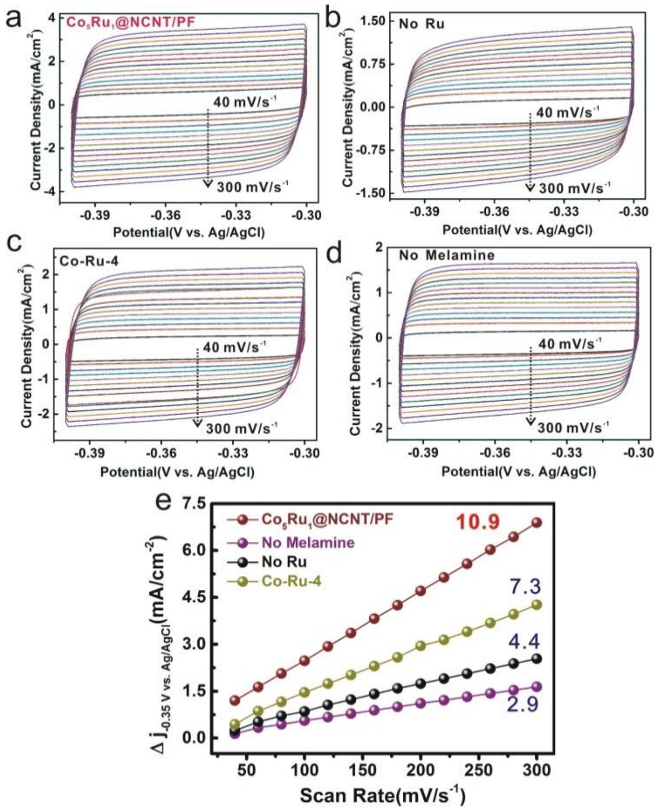

Figure S11. CV of the (a) Co_5Ru_1@NCNT/PFV(b) No Ru, (c) Co-Ru-4 and (d) no Melamine catalysts in 1 M KOH solution in the region of -0.4~-0.3 V vs. Ag/AgCl for HER. (e) The differences in current density variation (ΔJ=Ja-Jc) at an overpotential of -0.35 V plotted against the scan rate fitted to a linear regression enables the estimation of C_{dl}.

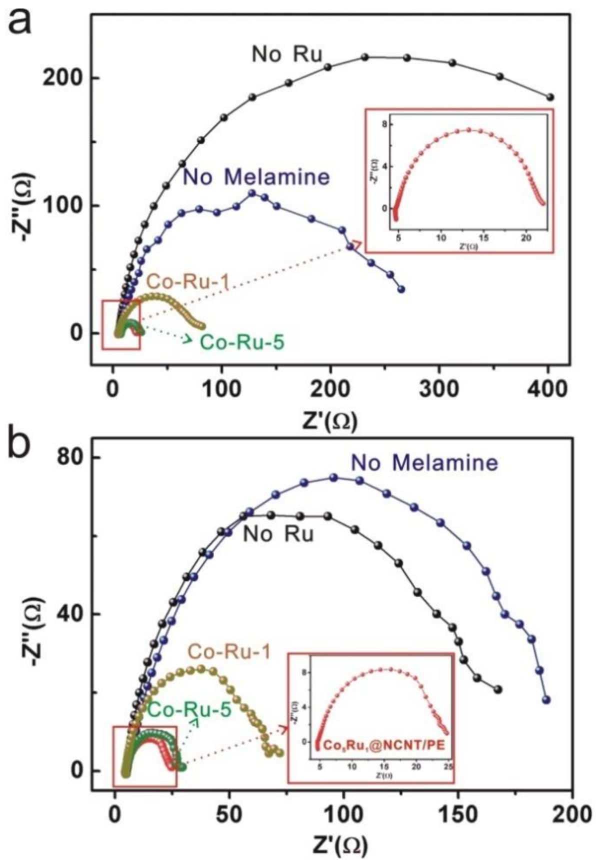

Figure S12. Electrochemical impedance spectroscopy (EIS) spectra of different catalysts in N_2-N_2-saturated (a) 1 M KOH and (b) 0.5 M H_2SO_4 solutions, respectively.

4. Supporting Tables

Table S1. The samples were prepared in similar procedures described above. The amount of ruthenium(III)nitrosyl nitrate was changed in following table.

No.	Weight Ratio$_{nominal}$ of Ru$_{precursor}$/ZIF-67	Ratio of Co/Ru (by ICP-MS)
Co@C(No Ru)	0	Co
Co-Ru-1	1:10	$Co_{12}Ru_1(Co_{0.93}Ru_{0.07})$
Co-Ru-2	1:5	$Co_9Ru_1(Co_{0.90}Ru_{0.10})$
Co-Ru-3	7:20	$Co_7Ru_1(Co_{0.87}Ru_{0.13})$
Co_5Ru_1@NCNT/PF	1:2	$Co_5Ru_1(Co_{0.83}Ru_{0.17})$
Co-Ru-4	3:4	$Co_7Ru_3(Co_{0.72}Ru_{0.28})$
Co-Ru-5	1:1	$Co_3Ru_2(Co_{0.62}Ru_{0.38})$

* Ru precursor: ruthenium(III) nitrosyl nitrate
Actual loading ratios of Co/Ru catalysts were measured by ICP-MS

Table S2. Structural parameters extracted from the Ru K-edge Ru χ(R) space spectra fitting of different catalysts.

Ru Co-Ru-1	Reduced Chi-square (χ_v^2)	R-factor (%)	amp/S_0^2	N$_{(Ru-Co\ path)}$	R$_{(Ru-Co\ path)}$ (Å)	$\sigma^2_{(Ru-Co\ path)}$ (10^{-3} Å^2)	ΔE_0 (eV)
	957.29	0.0284	0.82+/-0.11	10.0	2.569±0.054	2.7+/-1.8	4.89+/1.57

Ru Co_5Ru_1 @NCNT/PF	Reduced Chi-square (χ_v^2)	R-factor (%)	amp/S_0^2	N$_{(Ru-Co\ path)}$	R$_{(Ru-Co\ path)}$ (Å)	$\sigma^2_{(Ru-Co\ path)}$ (10^{-3} Å^2)	ΔE_0 (eV)
	948.70	0.0302	0.80+/-0.15	10.0	2.571±0.088	4.7+/-2.1	4.78+/0.66

	Reduced Chi-square (χ_v^2)	R-factor (%)	amp/S_0^2	$N_{(Ru\text{-}Co\ path)}$	$R_{(Ru\text{-}Co\ path)}$ (Å)	$\sigma^2_{(Ru\text{-}Co\ path)}$ (10^{-3}Å^2)	ΔE_0 (eV)
Ru Co-Ru-5	1071.26	0.0521	0.82+/-0.17	6.0	2.636± 0.066	5.7+/-3.1	6.63+/1.28
			amp/S_0^2	$N_{(Ru\text{-}Ru\ path)}$	$R_{(Ru\text{-}Ru\ path)}$ (Å)	$\sigma^2_{(Ru\text{-}Ru\ path)}$ (10^{-3}Å^2)	ΔE_0 (eV)
			0.85+/-0.15	4.0	2.6591 ± 0.082	2.6+/-0.8	2.58+/1.06

Table S3. Structural parameters extracted from the Co K-edge Co χ(R) space spectra fitting of different catalysts

	Reduced Chi-square (χ_v^2)	R-factor (%)	amp/S_0^2	$N_{(Co\text{-}C\ path)}$	$R_{(Co\text{-}C\ path)}$ (Å)	$\sigma^2_{(Co\text{-}C\ path)}$ (10^{-3}Å^2)	ΔE_0 (eV)
Co Co-Ru-1	5415.41	0.0582	0.81+/-0.15	2.0	1.790±0.041	4.2+/-1.3	1.83+/1.34
			amp/S_0^2	$N_{(Co\text{-}Co\ path)}$	$R_{(Co\text{-}Co\ path)}$ (Å)	$\sigma^2_{(Co\text{-}Co\ path)}$ (10^{-3}Å^2)	ΔE_0 (eV)
			0.84+/-0.14	9.0	2.391±0.055	5.7+/-2.4	2.79+/1.88
			amp/S_0^2	$N_{(Co\text{-}Ru\ path)}$	$R_{(Co\text{-}Ru\ path)}$ (Å)	$\sigma^2_{(Co\text{-}Ru\ path)}$ (10^{-3}Å^2)	ΔE_0 (eV)
			0.77+/-0.19	3.0	2.606±0.064	7.2+/-3.7	4.33+/2.56
			amp/S_0^2	$N_{(Co\text{-}Ru\text{-}Co\ path)}$	$R_{(Co\text{-}Ru\text{-}Co\ path)}$ (Å)	$\sigma^2_{(Co\text{-}Ru\text{-}Co\ path)}$ (10^{-3}Å^2)	ΔE_0 (eV)
			0.77+/-0.19	3.0	4.708±0.088	7.2+/-3.7	4.33+/2.56

	Reduced Chi-square (χ_ν^2)	R-factor (%)	amp/S_0^2	$N_{(Co\text{-}C\ path)}$	$R_{(Co\text{-}C\ path)}$ (Å)	$\sigma^2_{(Co\text{-}C\ path)}$ (10^{-3}Å^2)	ΔE_0 (eV)
Co Co_5Ru_1@NCNT/PF	5459.47	0.0543	0.84+/-0.11	2.0	1.682±0.027	3.0+/-1.9	3.11+/1.25
			amp/S_0^2	$N_{(Co\text{-}Co\ path)}$	$R_{(Co\text{-}Co\ path)}$ (Å)	$\sigma^2_{(Co\text{-}Co\ path)}$ (10^{-3}Å^2)	ΔE_0 (eV)
			0.82+/-0.18	8.0	2.391±0.039	4.6+/-2.8	4.79+/1.66
			amp/S_0^2	$N_{(Co\text{-}Ru\ path)}$	$R_{(Co\text{-}Ru\ path)}$ (Å)	$\sigma^2_{(Co\text{-}Ru\ path)}$ (10^{-3}Å^2)	ΔE_0 (eV)
			0.78+/-0.20	4.0	2.606±0.044	7.6+/-3.1	5.32+/3.02
			amp/S_0^2	$N_{(Co\text{-}Ru\text{-}Co\ path)}$	$R_{(Co\text{-}Ru\text{-}Co\ path)}$ (Å)	$\sigma^2_{(Co\text{-}Ru\text{-}Co\ path)}$ (10^{-3}Å^2)	ΔE_0 (eV)
			0.78+/-0.20	4.0	4.724±0.075	7.6+/-3.1	5.32+/3.02

	Reduced Chi-square (χ_v^2)	R-factor (%)	amp/S_0^2	$N_{(Co\text{-}C\ path)}$	$R_{(Co\text{-}C\ path)}$ (Å)	$\sigma^2_{(Co\text{-}C\ path)}$ (10^{-3}Å^2)	ΔE_0 (eV)
Co<br>Co-Ru-5	5371.26	0.0421	0.85+/-0.12	2.0	1.737±0.012	4.0+/-1.2	3.21+/1.79
			amp/S_0^2	$N_{(Co\text{-}Co\ path)}$	$R_{(Co\text{-}Co\ path)}$ (Å)	$\sigma^2_{(Co\text{-}Co\ path)}$ (10^{-3}Å^2)	ΔE_0 (eV)
			0.83+/-0.17	10.0	2.361±0.016	5.5+/-1.3	3.42+/2.01
			amp/S_0^2	$N_{(Co\text{-}Ru\ path)}$	$R_{(Co\text{-}Ru\ path)}$ (Å)	$\sigma^2_{(Co\text{-}Ru\ path)}$ (10^{-3}Å^2)	ΔE_0 (eV)
			0.78+/-0.20	2.0	2.655±0.034	7.2+/-3.7	5.05+/2.99
			amp/S_0^2	$N_{(Co\text{-}Ru\text{-}Co\ path)}$	$R_{(Co\text{-}Ru\text{-}Co\ path)}$ (Å)	$\sigma^2_{(Co\text{-}Ru\text{-}Co\ path)}$ (10^{-3}Å^2)	ΔE_0 (eV)
			0.78+/-0.20	2.0	4.751±0.032	7.2+/-3.7	5.05+/2.99

Table S4. Summary of some recently reported representative HER electrocatalysts in alkaline and acidic electrolytes.

**Catalyst**	**Current density (mA·cm^{-2})**	**Overpotential (1M KOH/mV)**	**Overpotential (0.5M H_2SO_4/mV)**	**Reference**
**Co_5Ru_1@NCNT/PF**	**10**	**28**	**45**	**This work**
	**100**	**128**	**108**	
CoRu@N-graphene	10	28	--	*Nat.Commun.***2017**, 8,14969[9]
	100	218	--	
$CoRu_{0.5}$/carbon quantum dots	10	18	--	*Angew. Chem. Int. Edit.* **2020**, DOI:10.1002/anie.202013985[10]
	100	about 215	--	
$Ru/C_3N_4/C$	10	79	70	*J. Am. Chem. Soc.***2016**, *138*, 16174-16181[11]
	20	about 100	about 90	
Ru@CN	10	50	126	*Energy Environ. Sci.***2018**, *11*, 800-806[12]
	60	about 145	about170	
Ru@NC (2.0 Ruwt%)	10	26	--	*Angew. Chem. Int. Edit.***2018**, *57*, 5848-5852[13]
	100	100	--	
Co-N-graphene	10	--	147	*Nat. Commun.***2015**, *6*, 8668(1-8)[14]
	20		about ~ 195	
CoNX/C	10	170	133	*Nat. Commun.***2015**, *6*, 7992(1-8)[15]
	100	--	--	
Co@N-CNTs@rGO	10	108	87	*Adv. Mater.***2018**, *30*, 1802011(1-10)[16]
		about ~ 200	--	

"--" stands for not given

Table S5. Comparison of electrocatalytic HER activity of Co_5Ru_1@NCNT/PFwith recently reported transition metal-based catalysts in neutral media.

Catalyst	Current density (mA·cm^{-2})	Overpotential (1M PBS/mV)	Durability (Time/Retention)	Reference
**Co_5Ru_1@NCNT/PF**	**10**	**28**	**24 h @30 mA·cm^{-2}**	**This work**
	**100**	**187**		
Co/N-doped carbon	10	163	70 h @10 mA·cm^{-2}	*Energy Environ. Sci.*, **2021**, 10.1039/D1EE00052G[17]
	80	about 320		
RhCu	10	57	12 h @10 mA·cm^{-2}	*Adv. Energy Mater.*, **2020**, *10*, 201903038[18]
	100	---		
MoP	10	196	2 h @10 mA·cm^{-2}	*ACS Catal.*, **2019**, *9*, 8712-8718[19]
	100	about 230		
RuCo@C	10	60	---	*J. Mater. Chem. A*, **2020**, *8*, 12810–12820[20]
	90	about 350		
Mo-WC@NCS	10	221	12 h @10 mA·cm^{-2}	*Nano Energy*, **2020**, *74*, 104850[21]
	100	---		
MoP NA/CC	10	187	46 h @10 mA·cm^{-2}	*Appl. Catal. B: Environ.*, **2016**, *196*, 193-198[22]
	100	---		
$Ni_{0.85}Se$@NC	10	183	10 h @10 mA·cm^{-2}	*Small*, **2020**,*16*, 2004231[23]
	100	---		
$NiFe_2O_4$/NF	10	197	---	*Nano Energy*, **2017**, *40*, 264-273[24]
	100	---		
CoO/Co_4N/NF	10	145	50 h @10 mA·cm^{-2}	*J. Mater. Chem. A*, **2018**, *6*, 24767-24772[25]
	100	---		

--" stands for not given

Table S6. Adsorption free energies for the adsorbed *H.

Catalyst	Adsorption free energy of *H (eV)	Adsorption site
Co(0001)	0.26	Co
(Subsurface)RuCo(0001)	0.20	Co
(Subsurface)RuCo(0001)@C/gN	0.14	Co
Ru(0001)	-0.06	Ru

Table S7. Kinetic free energy barriers for the Volmer step and the corresponding electron transfer coefficient α calculated from the experimental Tafel slopes.

Catalyst	Tafel slope (mV/dec)	$\Delta G^{\neq}$ (eV)	Transfer coefficient (α)
Co(0001)	120	0.75	0.49
RuCo(0001)	135	0.73	0.44
RuCo(0001)@C/gN	65	0.71	0.91
Ru(0001)	85	0.74	0.70

5. Supporting References

[1] G. Kresse, J. Hafner, *Phys. Rev. B* **1993**, *47*, 558.

[2] G. Kresse, J. Furthmüller, *J. Comput. Mater. Sci.* **1996**, *6*, 15.

[3] G. Kresse, J. Furthmüller, *Phys. Rev. B* **1996**, *54*, 11169.

[4] Y J. P. Perdew, K. Burke, M. Ernzerhof, *Phys. Rev. Lett.* **1996**, *77*, 3865.

[5] G. Kresse, D. Joubert, *Phys. Rev. B* **1999**, *59*, 1758.

[6] A. Taylor, R. W. Floyd, *Acta Crystallographica* **1950,** *3*, 285.

[7] V. A. Finkel, M. I. Palatnik, G. P. Kovtun, *Phys. Met. Metall.* **1971,** *32*, 231.

[8] S. B. Qadri, T. M. Keller, M. Laskoski, C. A. Little, M. S. Osofsky, H. R. Khan, *Appl. Phys. Lett.* **2007**, *91*, 3.

[9] J. Su, Y. Yang, G. Xia, J. Chen, P. Jiang, Q. Chen, *Nat. Commun.* **2017**, *8*, 14969.

[10] W. Li, Y. Zhao, Y. Liu, M. Sun, G. I. N. Waterhouse, B. Huang, K. Zhang, T. Zhang, S. Lu, *Angew. Chem. Int. Edit.* **2021**, *60*, 3290.

[11] Y. Zheng, Y. Jiao, Y. Zhu, L. H. Li, Y. Han, Y. Chen, M. Jaroniec, S.-Z. Qiao, *J. Am. Chem. Soc.* **2016**, *138*, 16174.

[12] J. Wang, Z. Wei, S. Mao, H. Li, Y. Wang, *Energy Environ. Sci.* **2018**, *11*, 800-806.

[13] Z.L. Wang, K. Sun, J. Henzie, X. Hao, C. Li, T. Takei, Y.M. Kang, Y. Yamauchi, *Angew. Chem. Inter. Edit.* **2018**, *57*, 5848.

[14] H. Fei, J. Dong, M. J. Arellano-Jiménez, G. Ye, N. Dong Kim, E. L. G. Samuel, Z. Peng, Z. Zhu, F. Qin, J. Bao, M. J. Yacaman, P. M. Ajayan, D. Chen, J. M. Tour, *Nat. Commun.* **2015**, *6*, 8668.

[15] H.-W. Liang, S. Brüller, R. Dong, J. Zhang, X. Feng, K. Müllen, *Nat. Commun.* **2015**, *6*, 7992.

[16] Z. Chen, R. Wu, Y. Liu, Y. Ha, Y. Guo, D. Sun, M. Liu, F. Fang, *Adv. Mater.* **2018**, *30*, 1802011.

[17] Z. Chen, H. Qing, R. Wang, R. Wu, *Energy Environ. Sci.* **2021**. doi:10.1039/D1EE00052G (2021)

[18] D. Cao, H. X. Xu, D. J. Cheng, *Adv. Energy Mater.* **2020**, *10*, 12.

[19] X. Xie, M. Song, L. Wang, M. H. Engelhard, L. Luo, A. Miller, Y. Zhang, L. Du, H. Pan, Z. Nie, Y. Chu, L. Estevez, Z. Wei, H. Liu, C. Wang, D. Li, Y. Shao, *ACS Catal.* **2019**, *9*, 8712.

[20] F. Zhang, Y. Zhu, Y. Chen, Y. Lu, Q. Lin, L. Zhang, S. Tao, X. Zhang, H. Wang, *J. Mater. Chem. A* **2020**, *8*, 12810.

[21] L. Wang, Z. Li, K. Wang, Q. Dai, C. Lei, B. Yang, Q. Zhang, L. Lei, M. K. H. Leung, Y. Hou, *Nano Energy* **2020**, *74*, 104850.

[22] Z. Pu, S. Wei, Z. Chen, S. Mu, *Applied Catalysis B: Environmental* **2016**, *196*, 193.

[23] Z. D. Huang, B. Xu, Z. G. Li, J. W. Ren, H. Mei, Z. N. Liu, D. G. Xie, H. B. Zhang, F. N. Dai, R. M. Wang, D. F. Sun, *Small* **2020**, *16*, 11.

[24] J. Liu, D. Zhu, T. Ling, A. Vasileff, S.-Z. Qiao, *Nano Energy* **2017**, *40*, 264.

[25] R.Q. Li, P. Hu, M. Miao, Y. Li, X.F. Jiang, Q. Wu, Z. Meng, Z. Hu, Y. Bando, X.-B. Wang, *J. Mater. Chem. A* **2018**, *6*, 24767.
